# Controversies in the Definition and Treatment of Idiopathic Short Stature (ISS)

**DOI:** 10.4008/jcrpe.v1i3.53

**Published:** 2009-02-01

**Authors:** Stefania Pedicelli, Emanuela Peschiaroli, Enrica Violi, Stefano Cianfarani

**Affiliations:** 1 ‘Rina Balducci’ Center of Pediatric Endocrinology, Department of Public Health and Cell Biology, Tor Vergata University, Rome, Italy; +39−06 72596178+39−06 5917415stefano.cianfarani@uniroma2.it‘Rina Balducci’ Center of Pediatric Endocrinology, Department of Public Health and Cell Biology, Room E−178, Tor Vergata University, Via Montpellier 1, 00133, Rome, Italy

**Keywords:** growth, Idiopathic short stature, growth hormone, insulin−like growth factor

## Abstract

The term idiopathic short stature (ISS) refers to short children with no identifiable disorder of the growth hormone (GH)/insulin like growth factor (IGF) axis and no other endocrine, genetic or organ system disorder. This heterogeneous group of short children without GH deficiency (GHD) includes children with constitutional delay of growth and puberty, familial short stature, or both, as well as those with subtle cartilage and bone dysplasias. In rare cases, ISS is due to IGF molecular abnormalities. In this review we tackle the major challenges in the definition and treatment of ISS.

**Conflict of interest:**None declared.

## DEFINITION

**A heterogeneous population**

Idiopathic short stature (ISS) is defined as a condition characterized by a height more than 2 standard deviations below the corresponding average height for a given age, sex and population, without findings of disease.(1, 2, 3) According to this definition, it is estimated that approximately 80% of all children referred for short stature, at the end of the diagnostic work−up, will be labeled as ISS.(4) The concept behind this definition is that ISS can be considered as part of the continuum extending from complete growth hormone deficiency (GHD) to normality and covering different degrees of GH secretion and responsiveness.

To better define this population, ISS has recently been subdivided into two major groups: a) familial short stature (FSS), when the child is short compared with the reference population, but remains within the range of target height; b) non−familial short stature (NFSS), when the child is short both in comparison with the reference population and the target height. This latter subgroup inevitably includes short children with constitutional delay of growth and puberty (CDGP).(1)

The need of a sub−categorization stems from the classical distinction between the “normal variants” of growth (namely FSS and CDGP), characterized by the achievement of an adult height within the range of target height, and the “real” ISS children, whose growth pattern and natural history are different from FSS and CDGP and are thought to have a different etiology.

**More about the distinction between ISS and normal variants of growth**

Classically, FSS children present with stature within the target height range, no bone age delay and normal growth rate (≥25^th^ centile), whilst CGDP subjects show short stature, bone age delay (usually 32 years), delayed onset of puberty (313 years in girls, ≥14 years in boys), normal growth rate (≥25^th^ centile), and often familial history of delayed puberty. 

It has been objected that before the age of 13 (for girls) or 14 (for boys) years, certainty about the timing of pubertal onset (normal or delayed) cannot be obtained. Before that age, a delayed bone age is indeed to some extent predictive for CDGP, but it is a clinical observation that some children with delayed bone age may enter puberty at a normal age, and vice versa. Therefore a clear distinction between ISS and CDGP may be difficult.(5) Nevertheless, provided that the child fulfills the criteria for the definition of CDGP and endocrine tests are normal, the likelihood of CDGP is extremely high. Moreover, typically the child with CDGP starts losing centiles in the growth curve around the age when puberty usually begins in the general population.

We believe that for the management of the child with short stature, the auxological sub−classification is helpful. With respect to the diagnostic process, in a child with FSS (if pathological causes of parental shortness are considered unlikely) the chance of finding a pathological disorder is low. Thus, the full set of diagnostic investigations may not be necessary, unless growth rate slows down, and these children attain their genetic potential. If a child’s height SDS is lower than the target range (NFSS) and there is a positive family history of CDGP, the likelihood of CDGP is high, and the experienced clinician may follow an expectant course.

Therefore, ISS is a diagnosis that is not based on positive findings in the diagnostic work−up, but on exclusion of other recognizable conditions. More specifically, children with ISS should be considered GH sufficient, should have normal body proportions, no history of a low birth size (small for gestational age, SGA), no chromosomal abnormalities, no dysmorphic syndromes and no systemic, endocrine or nutritional diseases.(1) Finally, normal variants of growth (FSS and CGDP) should be excluded. The completeness of the medical history (Table 1), the accuracy of the physical examination (Table 2) and the investigations (Table 3) determine the possibility of identifying underlying pathologies.

**The frequency of ISS amongst short children**

In short individuals the prevalence of known growth disorders is obviously much higher than in the total population, but these still constitute the minority. In most short children no diagnosis can be made, and these are labeled as having ‘‘idiopathic short stature”.

The percentage of pathology found in most studies is approximately 5%.(4, 6, 7) Whereas in 15% a history of a low birth weight or length for gestational age (SGA) is found.(8) This means that in approximately 80% of the short children presented to a pediatric clinic there is no history of a low birth weight and/or length, and no pathology can be detected. The vast majority of them have normal variants of growth (FSS or CDGP) and will attain an adult height within the target height range.

**Short stature: a statistical concept or a disease?**

Shortness is defined as a condition in which the height of the individual is 2 SD below the corresponding mean height for a given age, sex and population group.^1^ Therefore, short stature is defined on the basis of a statistical cut−off point which does not automatically imply the presence of an underlying pathology. Height distributes in a definite population according to a Gaussian curve in which subjects with a height <−2 SD can either be considered as the necessary 2.3% shortest part of the normal distribution, or as individuals with a disorder that restricts growth.(5)

**Pathophysiology of ISS**

ISS children represent a highly heterogeneous population with multiple potential pathophysiological mechanisms.(9)

In the diagnostic work−up for ISS, dysmorphic syndromes must be excluded, but it is not agreed how far genetic testing should go before the condition can be labeled as idiopathic. For example, there is wide agreement that all girls should be tested for Turner syndrome, but there is no consensus of whether all short children should be tested for a heterozygous deletion or mutation of SHOX, which has been described in about 2.5% of ISS children.(10) Recently a clinical score was developed to predict the likelihood of a SHOX defect and this can be used for improved selection of patients for testing.(11) There is no agreement on either what skeletal dysplasias should be excluded or what cut−off limit with respect to body proportions should be used. To assess body proportion, in Europe the sitting height/height (SH/H) ratio and in the United States the upper/lower segment ratio is used, but both of these ratios are strongly influenced by secular trend and reliable references are not available.

**Figure 1:**
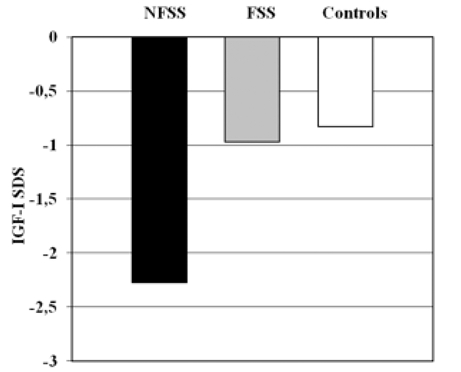
Mean serum IGF−I SDS concentration between NFSS (Non Familial Short Stature: height ≤−2 SDS and corrected target ≤−1.9 SDS), FSS ( Familial Short Stature: height ≤−2 SDS and corrected target >−1.9 SDS) and control group (height >−2 SDS). Data are expressed in mean ± SEM. Personal unpublished data.

By definition, children with ISS have normal GH secretion, which is almost always investigated by a pharmacological GH provocation test. Theoretically, they could have reduced spontaneous 24−hr GH secretion. However, the assessment of spontaneous GH secretion is very rarely included in the clinical evaluation of short stature and the entity of GH neurosecretory dysfunction (12) still remains controversial. Some ISS children have been found to have low concentrations of growth hormone binding protein (GHBP), suggesting reduced GH receptor (GHR) function.(13) These patients tend to have lower IGF−I levels yet higher endogenous GH secretion, suggestive of partial GH insensitivity (GHI).(14) An overlap exists between ISS patients and those with partial or atypical GHI. This was appreciated from the study of the European cohort of GHI patients, (15) some of whom did not have the typical appearance of Laron syndrome. In fact, some had milder short stature and normal facial appearance with less biochemical abnormalities.(16) However, it has to be pointed out that the vast majority of ISS children have IGFI in the low normal range and normal GH concentrations. Our preliminary data, collected from a group of 94 children (subdivided in NFSS, FSS and controls), show a significant difference in mean serum IGF−I SDS concentrations between NFSS children and controls (Figure 1). The existence of a mild form of GHI is hence extremely rare in ISS population.

Defects in post−receptor GH signaling have been described.(17, 18, 19) A homozygous mutation in exon 15 of the STAT5b gene has been reported. The mutant protein could not be activated by GH, therefore failing to activate gene transcription.(18) However, the first reported case showed a complex phenotype characterized by features of severe GHI together with immunodeficiency consistent with a non−functional STAT5b.

In summary, some children labeled as ISS may have a low spontaneous GH secretion that has not been detected, a genetic disorder that has not been studied (e.g. SHOX haploinsufficiency), a dysfunctional GH promoter or an abnormal GH molecule that has not been analyzed, or some form of decreased responsiveness to GH by a genetic defect in GH signaling. It is likely that in the coming years more identifiable clinical conditions will be discovered that nowadays are still resting under the cover of the term “idiopathic”.

**Table 1:**
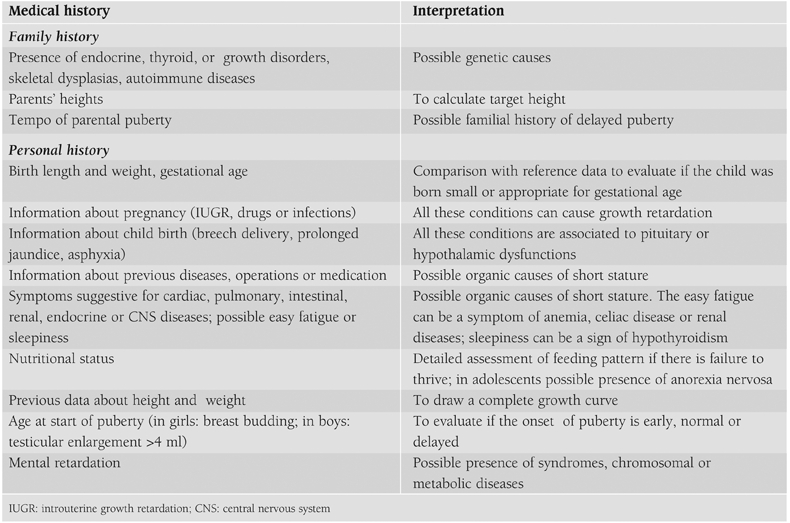
Information to be gathered about familial and personal history.

**Table 2:**
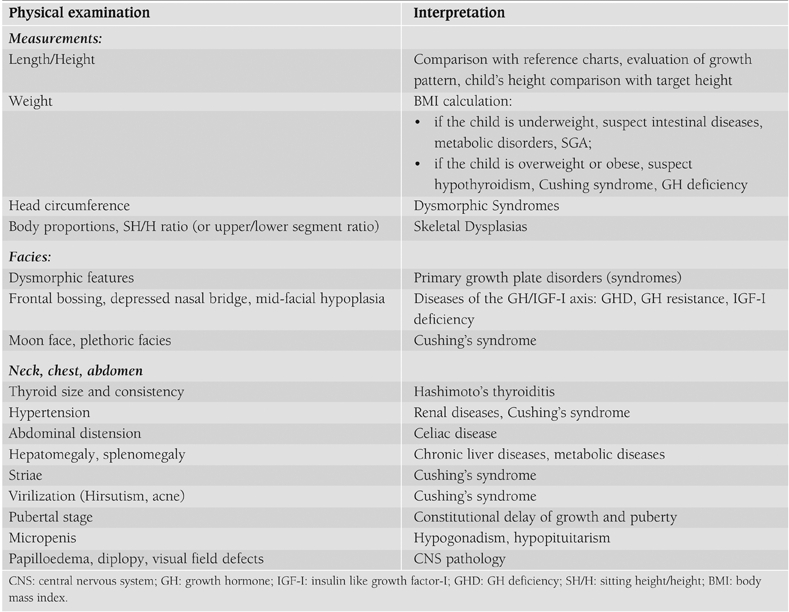
Signs and symptoms to look for in the clinical examination of a child with short stature.

**Table 3:**
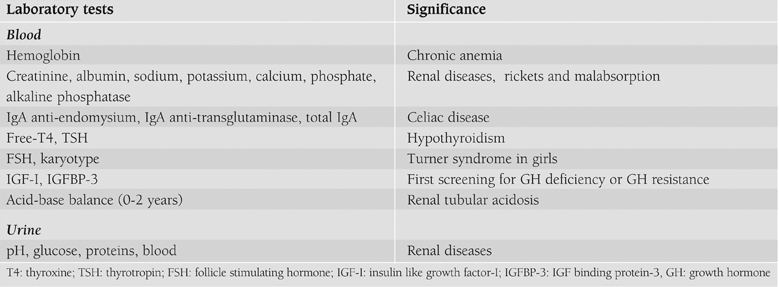
Laboratory tests for screening of children with short stature.

## THERAPY

**GH**

A careful review of the diagnostic approach to short stature over the last 50 years would inevitably lead to the conclusion that rather than diagnosis dictating therapy, the availability of GH dictated diagnosis. Since recombinant GH has become widely available (19), its indications are substantially increased. The treatment with GH for ISS has been approved by the Food and Drug Administration (FDA) in United States in July 2003 for children shorter than −2.25 SDS (1.2 percentile). Very few studies report on the efficacy and safety of long−term GH treatment of ISS children until the achievement on adult height, and, for this reason, in Europe this indication has not yet been approved.

According to the “Consensus guidelines for the diagnosis and treatment of children with ISS” this treatment should be considered if the height ranges from −2 SDS to −3 SDS and the optimal age to initiate treatment is 5 years to early puberty.(20) The therapy should generally not be started before the age of four and it should be prescribed until the growth is complete (i.e. growth rate less than 1−2 cm/years, and/or bone age over 16 years in boys and 14 years in girls).(2, 20) Alternatively the treatment could be discontinued when child’s height achieves the normal adult range (over −2 SDS). It is important to consider adverse effects, cost of therapy, patients’ expectations, ethical issues (21) and the impact of height gain on physical and psychosocial well−being.(20)

In the United States, the current FDAapproved doses for GH in ISS are up to 0.30–0.37 mg/kg • wk.(22) The dose may be increased if the growth response is considered inadequate, but there are no definitive data concerning the long−term safety of doses higher than 50 μg/kg/day (20), even if the upper limit of GH dosage used in other pediatric conditions is approximately 70 μg/kg/day.(23, 24) The evaluation of serum IGF−I levels, together with auxological parameters, is important to assess efficacy, safety and compliance and to adjust the dose, in particular in ISS patients in whom potential GHI may be the cause of a poor response to the standard dose. The correlation between IGF−I levels in prepubertal Ghnaive children with ISS and change in height SDS shows that higher growth response corresponds to lower baseline serum IGF−I levels.(25, 26) IGF−I levels that are consistently elevated (32.0 SDS) should prompt consideration of GH dose reduction.(20)

A successful response to GH treatment in the first year is defined by a delta height SDS more than 0.3 to 0.5, a first year height velocity increment more than 3 cm/year or a height velocity SDS more than 1, because height velocity in cm/yr depends on age and is less reliable. To evaluate the long−term response a number of parameters should be taken into account:(20) a) adult height SDS; b) height gain in SDS in comparison with the height at start of therapy; c) difference between adult height and predicted height; d) difference between the adult height and the target height.

GH treatment increases growth rate in the first year, but there are controversies about the effect on final height. It is difficult to obtain homogeneous results about the effect of treatment, because the ISS population is widely heterogeneous. Moreover a wide variety of dosages have been prescribed in several trials and most studies are uncontrolled. A recent systematic review has evaluated the short−term and long−term results of ten studies (seven of which were sponsored by or received support from pharmaceutical companies), published between 1989 and 2004, that included 741 children with ISS, treated with GH from 6 months to 6.2 years versus placebo or no treatment. Only one study reported adult height(27) and one reported near final height.(28) Near final height of treated girls was on average 7.5 cm higher than untreated controls corresponding approximately to 1.23 SDS.(28) Treated children showed on average an adult height 3.7 cm (or 0.51 SDS) higher than placebo−treated group.(27) These results are partially similar to those reported in a previous meta−analysis, that analyzed 10 controlled trials (some of which considered also by the Cochrane review) and 28 uncontrolled trials. In this report the adult height achieved by GH−treated individuals exceeded that of untreated controls by 0.78 to 0.84 SDS, equivalent to 5− 6 cm.(21) The National Cooperative Growth Study (a large surveillance study of GH use in the North America, in which 20% of all children had ISS), showed an increment of growth rate and height SDS during the first year of treatment, followed by lesser degrees of catch−up over the next 6 years. Nevertheless, in the 7th year of therapy mean growth rate of the children was greater than the pretreatment value.(29)

Recently, a randomized controlled trial in ISS children has investigated the effect on growth up to the achievement of adult height of two GH doses (33 or 67 μg/kg/day), demonstrating that GH treatment significantly increased the adult height by approximately 1 SDS (approx. 6 cm) after a mean duration of therapy of 5.9 ± 1.1 years, allowing them to attain a height close to that of their parents. The GH effect was dose−dependent and the children with parents of normal heights responded best.(30)

In attempt to find the predictive factors of growth response to GH therapy to identify the children that could benefit from GH therapy, several multivariate models have been proposed. The best growth response is found in children with parents of normal height (NFSS) compared with children from short parents (FSS).(27, 30, 31) Bone age delay is another important predictor,(27, 31) being positively related to the response. The prediction model of Leschek et al.(27) explains 84% of the variation in height gain and indicates that the best response is associated with lower baseline height SDS relative to gender adjusted midparental height SDS, lower pretreatment height velocity, lower baseline IGFI concentrations and greater bone age delay. Nevertheless if the variables were considered individually, only bone age delay was significantly correlated with the height gain.(27)

One of the aims of long−term GH treatment is the improvement of quality of life, but validated instruments to evaluate this effect are not currently recommended as part of routine care.(20) Short stature among children with ISS enrolled in the only longterm placebo−controlled study was not associated with problems in psychological adaptation or self−concept with the psychological instruments employed. GH treatment was associated with a trend toward improvement in problem behaviours, as measured by questionnaires completed by study participants’ parents. It remains to be determined whether GH treatment significantly impacts adaptation, psychosocial function, or quality of life in children with ISS.(32)

The possible side effects in GH−treated children with ISS are similar to those previously reported in children receiving GH therapy for other indications.(20)

Regular monitoring for scoliosis, tonsillar hypertrophy, papilledema and slipped capital femoral epiphysis should be performed as part of the regular physical exam during follow− up visits. Thus far, no instances of elevated blood glucose in GH−treated patients with ISS have been reported, but monitoring of glucose metabolism is strongly advised.

Increased risk of neoplasia or cancer relapses has been associated with GH therapy. Large−scale epidemiological studies have demonstrated a link between high concentrations of IGF−I and many of the common cancers of adulthood, such as carcinomas of prostate, lung, breast and colon.(33, 34, 35)Overall, available data suggest that high levels of IGF−I and low levels of IGF binding protein−3 (IGFBP−3) are predictive of an increased cancer risk. GH causes increased serum IGF−I and to a lesser extent, IGFBP−3 and consequently increases the ratio of IGFI to IGFBP−3, thus raising concern on safety of the long−term GH therapy.(36, 37, 38)

To date, no increased risk of malignancy in ISS children undergoing GH treatment has been reported. However, it should be recognized, that the available pharmacovigilance data is insufficient in several aspects: a) very few long−term randomized controlled trials have been conducted in ISS children so far; b) registration trials of GH indications have relied on limited number of patients, have not included randomized control or placebo groups (with a very small number of exceptions) and are not a reliable source of safety data when dealing with rare events; c) most of the available information has been obtained from large post−marketing studies organized by GH manufacturers and conducted for too limited time. Indeed these programs have efficiently identified health issues occurring during the treatment (diabetes, slipped capital femoral epiphyses, intracranial hypertension), but cannot address health issues occurring after treatment is discontinued; d) national pharmacovigilance programs are poorly efficient to detect rare events that would occur several years after the discontinuation of GH treatment and that are unlikely to be notified.

More studies are needed to detect whether children and young adults who have been treated with GH are at increased risk of cancer.

**The Role of GH Treatment Alternatives**

**Androgens**

In boys with CDGP, whose puberty and bone age are substantially delayed, testosterone is the appropriate therapy. Oxandrolone treatment was also proposed as a valid therapeutic approach in CDGP children. Although it offers the advantage of oral administration, the disadvantages are the weak androgenic activity and the remote risk of hepatotoxicity.

**GnRH analogues**

Therapy with GnRH analogues (GnRHa) has been proposed to delay bone maturation and pubertal development ultimately leading to increased adult height.(39, 40)

Monotherapy with GnRHa in both sexes has shown a small and variable effect on adult height gain and the duration of treatment seems to be positively correlated with the gain in final height. However, due to the lack of conclusive data, this treatment is not recommended. Concerns have been raised regarding potential adverse effects of GnRHa, including on bone mineral density (41) and on the psychological consequences of delaying puberty.(42) Combination therapy with GnRHa and GH might have potential value, although convincing data are not yet available.

## AROMATASE INHIBITORS

Aromatase inhibition has been proposed to stimulate growth in the presence of androgens, whereas bone age advancement is slowed due to inhibition of estrogen production. An increase in predicted adult height has been shown in males with CDGP,(43) but adult height data are not available. The use of aromatase inhibitors in females is currently considered unsafe. To date, the long−term efficacy and safety of aromatase inhibitors in males with ISS has not been demonstrated.

Combination therapy with aromatase inhibitors and GH has been reported to slow down the tempo of bone age acceleration and increase predicted adult height,(44) however, long−term follow−up of these patients is still required.

In conclusion, aromatase inhibitors might theoretically represent suitable therapeutic agents, alone or in combination with GH, in boys with ISS. Evidence, however, is still limited and this treatment should be still considered only within controlled clinical trials.

## IGF-I

In the United States, Japan, and Europe IGF−I has been approved for treatment of non−GH deficient children with severe IGF−I deficiency.(45) Therefore, IGF−I therapy might be theoretically considered in those ISS children who do not respond to GH and have low IGF−I concentrations. However, the finding of subnormal IGF−I levels in ISS children is exceptional. In subjects with IGF−I in the low normal range there is no controlled study comparing the effect of the therapy with GH or GH treatment alternatives with IGF−I administration.
